# Sodium Selenate Under Moderate Salinity Stress Enhances Selenium Concentration and Antioxidant Activity in Dill (*Anethum graveolens* L.) Across PFAL and Greenhouse Systems

**DOI:** 10.3390/plants15030502

**Published:** 2026-02-05

**Authors:** Cosimo M. Profico, Saeid Hazrati, Andrea Ertani, Silvana Nicola

**Affiliations:** 1 Department of Agricultural, Forest, and Food Sciences, Inhortosanitas Lab, University of Turin, 10095 Grugliasco, TO, Italy; cosimomatteo.profico@unito.it (C.M.P.); andrea.ertani@unito.it (A.E.); 2 Department of Health Sciences, School of Medicine, University of Piemonte Orientale, 28100 Novara, NO, Italy; 3 Department of Agronomy and Plant Breeding, Faculty of Agriculture, Azarbaijan Shahid Madani University, Tabriz 5375171379, Iran

**Keywords:** biofortification, aromatic plants, oxidative stress enzymes, selenium content, hydroponics

## Abstract

Enhancing selenium (Se) content of aromatic plants addresses micronutrient deficiencies affecting billions. Plants are the primary dietary Se source, so biofortification can enhance Se intake. This study examined the effects of Se biofortification with sodium selenate (5 μM Na_2_SeO_4_) and moderate salinity stress (10 mM sodium chloride NaCl) on dill (*Anethum graveolens* L.) grown in a Plant Factory with Artificial Lighting using Nutrient Film Technique (NFT-PFAL) or Floating System (FS-PFAL), and in a Greenhouse with FS (FS-GH). Se biofortification and moderate salinity stress did not affect dill yield in any hydroponic system. Plants under combined Se biofortification and salinity stress (Se + NaCl) showed increased Se concentration in leaves of 31.78 mg kg^−1^, 33.12 mg kg^−1^, and 23.32 mg kg^−1^ in NFT-PFAL, FS-PFAL, and FS-GH, respectively, compared to Se alone. Total phenolics content in leaves increased under Se biofortification with salinity stress across all systems, showing 159.57%, 223.13%, and 82.64% increases over control in NFT-PFAL, FS-PFAL, and FS-GH. Oxidative stress enzymes increased in response to Se, NaCl, and combined treatments across systems. FS-GH showed highest ascorbate peroxidase and guaiacol peroxidase activities, while PFAL systems showed lower but comparable activities. This study demonstrates that combining Se biofortification with moderate salinity stress in hydroponic systems can enhance plant functionality and human nutrition.

## 1. Introduction

Selenium (Se) is an essential micronutrient for animals and humans [1]. Se deficiency is reported to affect approximately 800 million (15%) people worldwide [2]. Biofortification with Se has emerged as a viable solution to reduce the deficiency of this element in the human diet by delivering precise doses to plants during their growth phase [3,4,5]. However, this practice requires careful implementation to prevent Se toxicity in plants and humans [6]. Among the various biofortification approaches, the agronomic strategy has been widely adopted, applying low Se concentrations to increase the concentration of this element in food crops while minimising potential toxicity risks [3]. Se uptake in plants occurs mainly via sulphate transporters located in the roots, since Se is absorbed predominantly as selenate (SeO_4_^2−^), a structural analogue of sulphate (SO_4_^2−^) [7,8]. This competitive interaction between Se and sulphur [8] is particularly critical in hydroponic systems where nutrient availability and ionic balance are precisely controlled [9]. Beyond direct competition for uptake, the Se-sulphur interaction influences broader physiological processes. Se biofortification may enhance antioxidant capacity through modulation of ROS signalling pathways, while sulphur metabolism plays a central role in osmotic adjustment under salinity stress [7,8]. In closed or recirculating hydroponic systems, understanding the relationship between selenate-sulphur antagonism and salinity is particularly important, since managing the nutrient solution directly impacts both crop quality and the risk of toxicity [7,10]. Once absorbed, Se is assimilated into organic compounds such as selenocysteine and selenomethionine. These selenoamino acids are crucial for both plant metabolism and human health [3,11]. Within plants, Se can enhance the biosynthesis of secondary metabolites, including phenolic compounds and flavonoids which have antioxidant properties contribute to the improvement of crop nutritional quality [12,13]. Several studies have demonstrated that low Se biofortification levels (5–10 μM) not only increase Se accumulation in plant tissues but also enhance nutritional quality and, in some cases, crop yield, in species such as *Anethum graveolens* L. [14], *Lactuca sativa* L. [15], *Spinacia oleracea* L. [16], and *Solanum lycopersicum* L. [17].

The rise in Se concentration in crops can be linked to the biofortification of this element, with its effectiveness further shaped by the specific agronomic biofortification technique used [18,19,20]. In their comprehensive review, Galić et al. (2021) [19] reported that the application of Se during the growth phase via foliar spraying improved Se concentration in plants and was more effective than soil-based biofortification techniques in many cases. Foliar application enables micronutrients to penetrate directly through the leaf cuticle and stomata, allowing their accumulation within the phloem. This pathway is considerably shorter than root uptake, potentially enhancing the efficiency of this method from a biofortification perspective [21]. Foliar Se application was chosen instead of nutrient solution enrichment to prevent competitive inhibition of sulphate uptake and to stop Se accumulation in recirculating hydroponic systems. This approach enables precise control of the dosage of Se while allowing interaction with root-zone salinity stress through phloem transport and long-distance signalling. This interaction is critical in PFAL and FS systems, where small solution volumes and high plant density increase sensitivity to ionic imbalances [8]. Due to its accessibility and simplicity, the foliar biofortification technique is gaining increasing popularity, especially in urban areas and regions where conventional farming is challenged by climate change [18,22,23].

Foliar Se biofortification can increase the concentration of this element in plants, allowing them to meet the recommended dietary allowance (RDA) for humans (40 µg per day) [24,25]. Se biofortification promotes plant growth and development, enhances antioxidant enzyme activity, and improves resistance to abiotic stresses, such as salinity. Shalaby et al. (2017) [26] reported that Se treatment of lettuce significantly increased the activity of oxidative stress related enzymes, reduced membrane damage, and improved yield under saline conditions compared with untreated plants.

Se plays a beneficial role in crop plants, particularly under abiotic stress conditions. Research by Shalaby et al. (2017) [26] and Shekari et al. (2017) [27] on *L. sativa* L. and *A. graveolens* L. demonstrated that moderate salinity stress (10 mM NaCl) could be optimal for enhancing antioxidant activity without inducing the adverse effects observed at higher salt concentrations. Furthermore, studies on *A. graveolens* L. and *Stachys byzantine* L. revealed that the combined application of Se biofortification and salinity stress increased antioxidant enzyme activity and improved overall plant growth [26,28]. Salinity stress typically leads to increased sodium (Na^+^) accumulation in leaves while reducing plant growth, cell membrane stability, and water uptake [29]. Therefore, the combined application of Se and moderate salinity stress can help modulate the plants ionic balance, specifically by improving the K^+^/Na^+^ ratio. This ratio is crucial for maintaining osmotic balance and mitigating salinity induced damage, as demonstrated in studies on *Cucumis sativus* L. [30] and *L. sativa* L. [28]. Se has also been shown to alleviate Na^+^ toxicity by preserving cell membrane functionality and increasing plant tolerance to adverse environmental conditions. These findings highlight the potential of combining Se foliar biofortification with moderate salinity stress to enhance Se in plants, while simultaneously, preventing excessive stress that could otherwise reduce yield and Se concentration.

To maintain plant health and ensure adequate Se concentration, it is essential to implement a system capable of controlling environmental conditions throughout the year. This approach supports the consistent production of crops with uniform quality [31]. Previous studies have emphasized the importance of providing growers with the ability to establish optimal growth conditions for crops, independent of outdoor weather variability [32]. Controlled Environment Agriculture (CEA) technology represents one of the most promising solutions, as it allows growers to overcoming the inherent simulations of conventional agricultural systems [31]. A key advantage of CEA technologies lies in their capacity to enable year-round crop production, irrespective of climatic fluctuations [32,33]. CEA systems primarily include greenhouses (GH) and Plant Factories with Artificial Lighting (PFAL). Greenhouses rely predominantly on natural sunlight, supplemented by systems that regulate temperature, humidity, and other environmental parameters essential for optimal plant development [32,33]. In contrast, PFAL utilize artificial light sources and precise environmental control, making them particularly suitable for urban environments or regions with limited natural resources [34,35]. CEA thus represents a promising strategy for producing fresh crops with enhanced and consistent nutritional quality compared to open-field cultivation [34,36]. Among the diverse approaches aimed at improving crop nutritional value within these systems, biofortification has attracted increasing attention due to its effectiveness in enhancing Se concentration in plants [4,15,16].

The dill plant (*A. graveolens* L.) holds significant importance due to its aromatic and medicinal properties, as well as its content of essential oils, phenolic compounds, vitamins, and minerals. Moreover, it presents strong potential for biofortification aimed at further enhancing its nutritional value [27,37]. This herb also exhibited remarkable tolerance to NaCl salinity levels up to 12 dS m^−1^ [27,38], making it suitable for a wide range of controlled cultivation systems and a promising candidate for growth under increasing salinity conditions in fresh and groundwater [39]. Growing dill in CEA systems represents a strategic opportunity to meet year-round consumer demand with standardised quality, optimised nutritional profiles, and ensuring standardized quality, optimized nutritional profiles, and reduced environmental impact particularly in regions with limited agricultural resources or challenging climatic conditions [14,40]. The literature robustly supports Se as a tool for mitigating salinity stress in a wide range of crops, including in hydroponic systems [41,42]. The mechanisms, antioxidant enhancement, osmotic adjustment, and ion homeostasis, are well established and likely to be relevant across CEA systems. However, the lack of system-specific studies means it is unclear whether these responses are modulated by the type of hydroponic technique: Nutrient film technique (NFT) and Floating system (FS) or by the broader environment (PFAL vs. GH). Most studies use generic hydroponic setups or soil-based systems, and few, if any, specify or compare NFT and FS directly. Similarly, while PFAL and GH systems are both used for CEA, no direct comparative studies under combined Se and salinity treatments were identified. This gap is significant, as environmental factors (e.g., light spectrum, humidity, air flow) and system design (e.g., root zone oxygenation, nutrient delivery) could influence Se uptake, distribution, and efficacy under salinity stress [43,44]. The evidence base is strong for the general benefits of Se under salinity, but system-dependence remains an open question. Some reviews caution that hydroponic results may not fully translate to soil or commercial-scale CEA systems [41,45]. The present study provides a comprehensive investigation of the effects of Se biofortification on dill as a high-value aromatic crop that is sensitive to stress and has measurable quality parameters. It is a short-cycle PFAL crop for which there is limited data on the interaction between selenium and salinity within the *Apiaceae* family. Specifically, this research uniquely compares plant yield and quality across two distinct CEA environments: a PFAL utilizing both the FS and NFT, and a GH employing the FS. This approach in this study enables the evaluation of Se biofortification efficacy across different cultivation systems and offers valuable insights into the development of optimized strategies to enhance crop performance, salinity tolerance, and Se concentration in emerging agricultural technologies.

## 2. Results

### 2.1. Yield and Dry Weight

The results for yield and DW are presented in Table 1 and Table 2. Within each cultivation system, no significant effects of Se and NaCl were observed on yield or DW. When comparing the systems, the yield did not differ significantly. In contrast, DW was significantly higher in FS-GH (188.72 mg g^−2^) than in NFT-PFAL (169.97 mg g^−2^) and FS-PFAL (166.55 mg g^−2^) (*p* ≤ 0.01, Table 2).

### 2.2. Total Chlorophyll Content

As reported in Table 1, total chlorophyll content (Chl) was significantly affected by the treatments in NFT-PFAL and FS-PFAL (*p* ≤ 0.001). In NFT-PFAL, NaCl showed the lowest value (2.47 mg g^−1^ FW), which was significantly lower than that of all other treatments. Se treatment resulted in an intermediate value (3.08 mg g^−1^ FW), which was lower than that of the control (4.24 mg g^−1^ FW) but higher than that of NaCl. Se + NaCl (3.36 mg g^−1^ FW) was intermediate, not differing significantly from Se, but still lower than the control.

In FS-PFAL, NaCl again showed the lowest value (2.48 mg g^−1^ FW) for Chl content. Se (3.08 mg g^−1^ FW) was intermediate, significantly lower than that in the control (5.47 mg g^−1^ FW) but higher than that in the NaCl treatment. Se + NaCl (3.94 mg g^−1^ FW) was also intermediate, significantly higher than Se but still lower than the control. In FS-GH, no significant differences were detected among the treatments.

According to Table 2, Chl differed among the systems (*p* ≤ 0.05). FS-GH recorded the highest mean value (4.41 mg g^−1^ FW), which was significantly higher than that of NFT-PFAL (3.29 mg g^−1^ FW). FS-PFAL (3.74 mg g^−1^ FW) showed intermediate values that did not differ significantly from either FS-GH or NFT-PFAL.

### 2.3. Total Phenolics Content

In all the cultivation systems, the treatments significantly influenced the total phenolics content (TPC) (*p* ≤ 0.001, Table 1).

In NFT-PFAL, the control plants showed the lowest value (1.85 nmol mg^−1^), Se was higher (3.28 nmol mg^−1^), and NaCl increased further (4.11 nmol mg^−1^), whereas Se + NaCl resulted in the maximum concentration (4.88 nmol mg^−1^).

In FS-PFAL, the control again recorded the lowest value (1.47 mM g^−1^ FW), Se was higher (2.79 nmol mg^−1^), while NaCl (4.66 nmol mg^−1^) and Se + NaCl (4.75 nmol mg^−1^) reached significantly higher values.

In FS-GH, TPC ranged from 3.62 nmol mg^−1^ in the control and 3.66 nmol mg^−1^ in Se to 5.14 nmol mg^−1^ in NaCl and 6.63 nmol mg^−1^ in Se + NaCl.

According to Table 2, no significant differences were observed among the systems.

### 2.4. Antioxidant Enzyme Activities

As shown in Table 1, guaiacol peroxidase (GPX), catalase (CAT), and ascorbate peroxidase (APX) activities were significantly affected by the treatments in all cultivation systems (*p* ≤ 0.001).

In NFT-PFAL, GPX was lowest in the control (95.65 μmol min^−1^ mg^−1^ protein) and highest (131.25 μmol min^−1^ mg^−1^ protein) in the Se + NaCl treatment, with Se and NaCl showing intermediate values. CAT followed the same pattern, from the lowest in the control (28.62 μmol min^−1^ mg^−1^ protein) to the maximum in the Se + NaCl treatment (41.77 μmol min^−1^ mg^−1^ protein). APX increased similarly, with the control being the lowest (34.21 μmol min^−1^ mg^−1^ protein) and Se + NaCl being the highest (46.33 μmol min^−1^ mg^−1^ protein); NaCl was significantly lower than Se + NaCl, whereas Se did not differ from NaCl.

In FS-PFAL, GPX was lowest in the control (131.29 μmol min^−1^ mg^−1^ protein) and highest in the Se + NaCl group (147.65 μmol min^−1^ mg^−1^ protein), with Se significantly lower than that in the Se + NaCl group, whereas NaCl did not differ. CAT activity increased from the control (28.59 μmol min^−1^ mg^−1^ protein) to the maximum in the Se + NaCl group (36.46 μmol min^−1^ mg^−1^ protein), with NaCl being higher than Se. APX was lowest in the control (34.10 μmol min^−1^ mg^−1^ protein) and reached the highest levels under NaCl and Se + NaCl (43.47 μmol min^−1^ mg^−1^ protein), both significantly higher than that under Se treatment.

In FS-GH, GPX ranged widely, from the lowest in the control (156.77 μmol min^−1^ mg^−1^ protein) to the maximum under NaCl (266.87 μmol min^−1^ mg^−1^ protein), with Se + NaCl (246.79 μmol min^−1^ mg^−1^ protein) also significantly higher than Se. CAT was lowest in the control (29.12 μmol min^−1^ mg^−1^ protein) and highest in the Se + NaCl group (43.04 μmol min^−1^ mg^−1^ protein), with NaCl not differing significantly from Se + NaCl but higher than Se. APX was lowest in the control group (43.16 μmol min^−1^ mg^−1^ protein) and highest in the Se + NaCl group (49.27 μmol min^−1^ mg^−1^ protein), with NaCl significantly higher than Se.

As shown in Table 2, significant differences among systems were observed for GPX and APX (*p* ≤ 0.001), whereas CAT did not vary significantly (ns). FS-GH recorded the highest mean values for both GPX (211.43 μmol min^−1^ mg^−1^ protein) and APX (46.49 mM g^−1^ FW) compared with NFT-PFAL (GPX: 112.77 μmol min^−1^ mg^−1^ protein; APX: 39.69 mM g^−1^ FW) and FS-PFAL (GPX: 121.54 μmol min−1 mg−1 protein; APX: 40.40 mM g^−1^ FW). CAT showed no significant differences among NFT-PFAL (34.08 μmol min^−1^ mg^−1^ protein), FS-PFAL (32.31 μmol min^−1^ mg^−1^ protein), and FS-GH (36.08 μmol min^−1^ mg^−1^ protein).

### 2.5. Selenium, Potassium, Sodium, and Magnesium

As reported in Table 3, the Se and sodium (Na^+^) concentrations were strongly affected by the treatments. In NFT-PFAL, the Se content was lowest in the control and NaCl (approximately 0.6 mg kg^−1^ DW) and increased markedly under Se (25.55 mg kg^−1^ DW) and Se + NaCl (31.78 mg kg^−1^ DW). The Na^+^ concentration was highest under NaCl (680.75 mg kg^−1^ DW), lowest in the control (437.20 mg kg^−1^ DW) and Se (388.75 mg kg^−1^ DW), with intermediate values under Se + NaCl (616.15 mg kg^−1^ DW). K^+^ content was reduced in NaCl and Se + NaCl compared to the control and Se. The magnesium (Mg^2+^) content did not vary significantly among the treatments.

In FS-PFAL, Se was lowest in the control (0.86 mg kg^−1^ DW) and NaCl (0.66 mg kg^−1^ DW) and increased under Se (23.67 mg kg^−1^ DW) and Se + NaCl (33.12 mg kg^−1^ DW). Na^+^ reached the highest levels under NaCl (595.88 mg kg^−1^ DW) and Se + NaCl (605.30 mg kg^−1^ DW) and the lowest under Se (337.03 mg kg^−1^ DW). K^+^ did not differ significantly, whereas Mg^2+^ increased under NaCl compared to the other treatments.

In FS-GH, Se was lowest in the control (0.70 mg kg^−1^ DW) and NaCl (0.60 mg kg^−1^ DW), and highest under Se (17.83 mg kg^−1^ DW) and Se + NaCl (23.32 mg kg^−1^ DW). The Na^+^ content reached maximum levels in NaCl (2427.12 mg kg^−1^ DW) and Se + NaCl (2367.34 mg kg^−1^ DW), whereas the control (1260.39 mg kg^−1^ DW) and Se (1060.52 mg kg^−1^ DW) showed significantly lower values. K^+^ and Mg^2+^ did not show significant variations among the treatments.

According to Table 4, Se concentration was significantly higher in NFT-PFAL (14.62 mg kg^−1^ DW) and FS-PFAL (14.58 mg kg^−1^ DW) than in FS-GH (10.61 mg kg^−1^ DW). Na^+^ was strongly increased in FS-GH (1778.84 mg kg^−1^ DW) compared to NFT-PFAL (530.71 mg kg^−1^ DW) and FS-PFAL (489.49 mg kg^−1^ DW). K^+^ was higher in NFT-PFAL and FS-PFAL than in FS-GH. The Mg^2+^ content was lowest in FS-GH (4.65 g kg^−1^ DW), while NFT-PFAL and FS-PFAL recorded higher levels (approximately 5.7 g kg^−1^ DW).

### 2.6. Nitrogen, Carbon and Sulphur Content

As shown in Table 3, the concentrations of nitrogen (N), carbon (C), and sulphur (S) were not significantly influenced by the treatments within any of the three cultivation systems. Values remained relatively stable across control, Se, NaCl, and Se + NaCl, with no consistent treatment effects.

According to Table 4, the differences among the systems were significant. The N content was highest in NFT-PFAL (6.10%) and FS-PFAL (5.87%), whereas FS-GH showed the lowest values (5.55%). C content did not differ significantly across the systems, remaining stable at approximately 39–40%. The S concentration was markedly higher in FS-GH (0.95%) than in NFT-PFAL (0.66%) and FS-PFAL (0.63%).

### 2.7. Cross-Associations of the Studied Parameters

**Figure 1:**
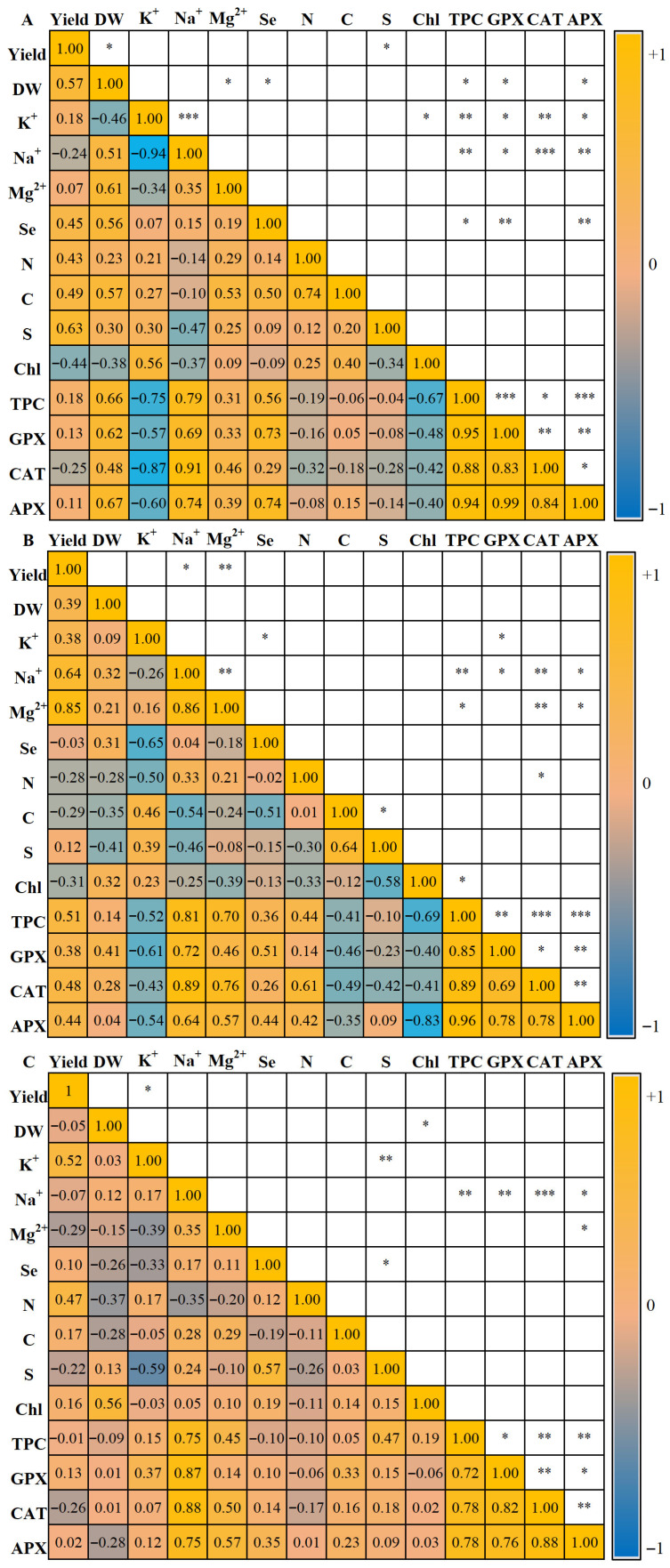
Pearson’s correlation coefficients among studied traits in Dill plants, with Se biofortification and under moderate salinity stress in NFT-PFAL (**A**), FS-PFAL (**B**), and FS-GH (**C**). The intensity of the colours indicates the strength of the correlation. Dry weight (DW), yield, potassium (K^+^), sodium (Na^+^), magnesium (Mg^2+^), selenium (Se), nitrogen (N), carbon (C), sulphur (S), chlorophyll (Chl), Total phenol Content (TPC), guaiacol peroxidase (GPX), catalase (CAT), and ascorbate peroxidase (APX). Asterisks denote statistically significant Pearson’s correlations * *p* < 0.05; ** *p* < 0.01; *** *p* < 0.001, while blank cells indicate non-significant correlations (*p* ≥ 0.05).

Figure 1 illustrates Pearson’s correlation matrices for dill plants grown in the three cultivation systems, highlighting trait-to-trait associations under Se biofortification, NaCl, and their combination.

In the NFT-PFAL system (Figure 1A), yield was positively correlated with DW and S. DW was also positively correlated with Na^+^, Mg^2+^, Se, C, TPC, GPX, and APX. Chl correlated positively with K^+^ content, whereas TPC showed positive associations with GPX, CAT, and APX. Among the enzymes, GPX correlated positively with CAT, and both GPX and APX were positively associated with DW, Na^+^, and TPC concentrations. Regarding nutrients, Na^+^ was positively correlated with Mg^2+^, TPC, GPX, CAT, and APX. Se was positively associated with DW, TPC, GPX, and APX levels, whereas N was positively correlated with C.

In the FS-PFAL system (Figure 1B), yield was positively correlated with Na^+^, Mg^2+^ and TPC. Chl showed a negative correlation with TPC. TPC was positively associated with Na^+^, Mg^2+^, GPX, CAT, and APX. For enzymes, GPX was positively correlated with CAT and APX, and negatively correlated with K^+^, Na^+^, and TPC. CAT and APX were strongly and positively correlated. Among the nutrients, K^+^ showed negative correlations with Se, N, TPC, GPX, and APX. Na^+^ was positively associated with Mg^2+^, TPC, GPX, CAT, and APX, but negatively associated with C. Mg^2+^ was positively correlated with yield, Na^+^, GPX, CAT, and APX. Se showed negative correlations with K^+^ and C, but a positive correlation with GPX. N was positively associated with CAT but negatively associated with K^+^. C correlated positively with S but negatively with Na^+^ and Se.

In the FS-GH (Figure 1C), yield correlated positively with K^+^, whereas DW correlated positively with Chl. TPC showed strong positive correlations with Na^+^, GPX, CAT, and APX. Among the enzymes, GPX was positively associated with Na^+^, TPC, CAT, and APX, whereas CAT was positively correlated with Na^+^, Mg^2+^, TPC, and APX. For nutrients, Na^+^ correlated positively with TPC, GPX, CAT, and APX. Mg^2+^ was positively associated with CAT and APX, whereas Se was positively correlated with S.

### 2.8. Principal Component Analysis

**Figure 2:**
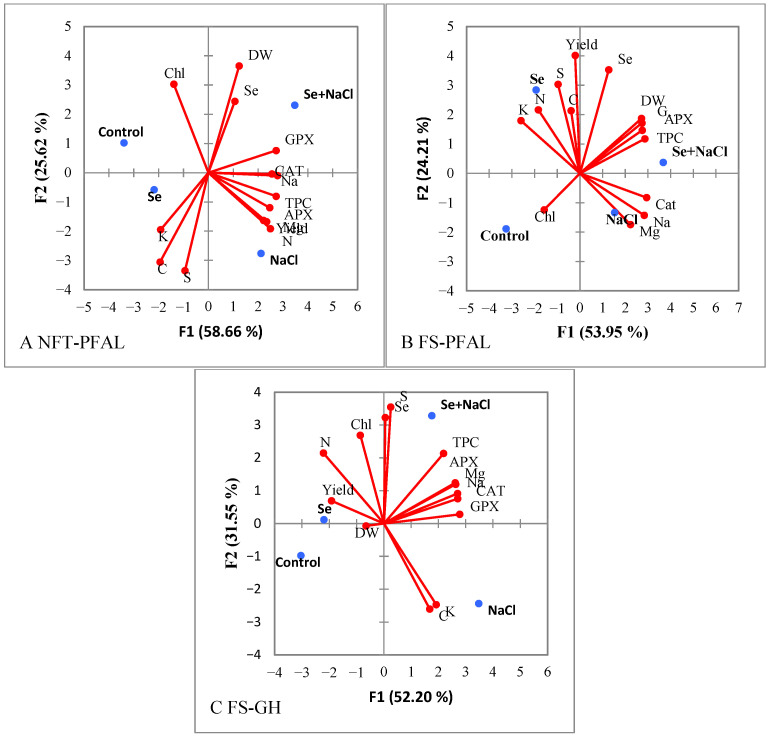
Principal component analysis of morphological and qualitative characteristics in dill plants, with Se biofortification and under moderate salinity stress in NFT-PFAL (**A**), FS-PFAL (**B**), and FS-GH (**C**). Dry weight (DW), yield potassium (K^+^), sodium (Na^+^), magnesium (Mg^2+^), selenium (Se), nitrogen (N), carbon (C), sulphur (S), chlorophyll (Chl), total phenol content (TPC), guaiacol peroxidase (GPX), catalase (CAT), and ascorbate peroxidase (APX).

PCA was performed to evaluate the morphological and qualitative traits of dill plants in response to Se, NaCl, and their combination (Se + NaCl) across the three cultivation systems (Figure 2).

In the NFT-PFAL system, PC1 explained 53.95% of the cumulative variance, and PC2 accounted for an additional 24.21% (Figure 2A). The Se + NaCl treatment was projected on the positive side of PC1 in the upper right quadrant, associated with higher DW, Se concentration, CAT, and GPX activity. NaCl treatment appeared on the negative side of PC2 in the lower right quadrant, linked with elevated Na^+^, CAT activity, and Mg^2+^. Se treatment was positioned on the positive side of PC1 in the upper left quadrant, aligned with higher N, K^+^, S, C, and yield.

In the FS-PFAL system, PC1 accounted for 58.66% of the cumulative variance, and PC2 explained 25.62% (Figure 2B). Se + NaCl was placed on the positive side of PC1 in the upper right quadrant, which was associated with greater DW, Se concentration, APX, and GPX. NaCl was positioned on the negative side of PC2 in the lower right quadrant, linked with higher Na^+^, CAT activity, N, yield, Mg^2+^, and TPC. Se treatment was projected on the negative side of PC2 in the upper left quadrant, which was associated with increased K^+^, S, and C concentrations.

In the FS-GH system, PC1 explained 52.20% of the cumulative variance, and PC2 accounted for 31.55% (Figure 2C). The Se + NaCl treatment was located on the positive side of PC1 in the upper right quadrant, associated with higher Na^+^, Mg^2+^, Se, S, TPC, APX, CAT, and GPX. NaCl treatment was positioned on the negative side of PC2 in the lower right quadrant, related to increased Na^+^ and K^+^. Se treatment was placed on the positive side of PC1 in the upper left quadrant, associated with greater yield, N, Se, and Chl.

## 3. Discussion

The objective of enhancing aromatic plants with Se holds considerable potential for improving mammal health and significantly enhancing the nutritional value of fresh horticultural products. To sustain the improved quality of these products, maintaining optimal production levels is of primary importance.

In terms of yield, our results demonstrated that Se biofortification combined with moderate salinity stress in dill grown in all three hydroponic systems did not negatively affect yield compared to the control plants. This observation was further supported by DW data, which revealed no significant differences among the three hydroponic systems. These findings are consistent with previous research by Hawrylak-Nowak (2015) [46] who reported that *L. sativa* L. subjected to Se biofortification and salinity stress (40 mM) maintained a higher yield than control plants. Similarly, Yaldiz & Camlica (2021) [47] demonstrated that *Salvia officinalis* L. grown under Se biofortification and salinity stress did not exhibit a reduced growth rate.

Our results differ from those of Shekari et al. (2017) [27], who reported a reduction in the Yield of dill under Se biofortification and salinity stress. This discrepancy could be due to their use of a higher salinity level (40 mM NaCl) compared to our use of a more moderate concentration (10 mM). This suggests that the significant yield reduction observed in their study was primarily due to severe salinity stress rather than Se supplementation. Our study and that of Shekari et al. (2017) [27], both studies administered the same Se dosage (5 μM). They noted no negative impact on yield, reinforcing the idea that appropriate Se supplementation can benefit plants under varying levels of salt stress. However, extreme salinity could surpass the protective effects of Se. In general, the effects of Se biofortification and salinity stress on plant growth can be either beneficial or detrimental, depending on the salinity dose applied. Salinity dose-dependent responses have been extensively documented in various plant species. Studies on *A. graveolens* L. [14], *Aloysia citrodora* Palau [48], *Lactuca sativa* L. [26], *Mentha* × *peppermint* L. [49], and *Salvia officinalis* L. [47], have demonstrated diverse responses to increasing salinity levels. These findings highlight the species-specific nature of salt tolerance mechanisms and the importance of considering varying salinity doses when evaluating plant responses to salt stress.

A compelling finding of this study was the significant difference in the cultivation time required for dill plants to reach the harvest stage across different cultivation systems. In the PFAL systems, the dill plants reached harvest maturity just 23 days after sowing, while in the GH environment, the harvest occurred 32 days after sowing. These nine days of differential data confirmed how optimised environmental management in indoor farming systems can substantially mitigate the adverse effects of external variables [50,51]. The harvest timing in both experiments was standardised based on the commercial stage, with plants harvested at an identical height of 25 cm across the three cultivation systems [52]. By avoiding the dependence on fluctuating solar radiation and elevated greenhouse temperatures [53], indoor systems ensure stable thermo-radiative conditions that accelerate plant development, as reflected by the markedly shorter cultivation cycle observed in this study [54].

The results demonstrated that Se biofortification and moderate salinity stress did not significantly affect the K^+^, C, N, and S concentrations in dill leaves. This is likely related to the Se biofortification and moderate salinity stress doses in these two experiments. Consistent with our findings, Sheikhi et al. (2024) [14] reported that in dill microgreens, K^+^ concentration remained unaffected by 1 mg L^−1^ (5 μM) Se biofortification. However, in contrast to our observations, Se biofortification increased the C and N concentrations in *Medicago sativa* L. plants [55]. The moderate salinity stress applied to dill plants in our study was apparently insufficient to induce changes in K^+^, N, and S concentrations, especially compared to the higher NaCl doses used in other studies investigating salinity tolerance effects in different plant species [56,57,58]. The Se concentration in dill leaves was significantly higher in both Se biofortification and Se + NaCl treatments in all three cultivation systems than in control leaves. In the PFAL system, the Se concentration was approximately 32 mg kg^−1^ DW (around 5.4 µg g^−1^ FW), whereas in the GH system it was 23 mg kg^−1^ DW (approximately 3.9 µg g^−1^ FW). Since dill is usually eaten in small amounts as a culinary herb, a typical serving of around 10 g of fresh dill would contain approximately 54 µg (PFAL) or 39 µg (GH) of Se, close to the recommended daily intake of 55 µg for adults. These levels are well below the tolerable upper intake level (UL) of 400 µg Se per day, indicating that the consumption of Se-biofortified dill in typical dietary amounts poses no risk of toxicity [5]. However, total Se intake should be evaluated in the context of an individual’s overall diet. These Se concentrations align with the finding reported by Mechora et al. (2017) [59], which demonstrated that Se biofortification with 1 mg L^−1^ on *Apium repens* L. has increased the Se concentration up to 35 mg kg^−1^ DW.

The findings from our experiments suggest that dill baby leaves can be enriched through Se foliar biofortification and salinity stress in both PFAL and GH systems. Subramanyam et al. (2019) [60] reported that under salt stress conditions, the application of sodium selenate can increase the concentration of Se in plant tissues. They attributed this to the adaptive response of the plant, which could favour the uptake or concentration of the micronutrient in residual tissues. However, excess salinity can limit Se uptake and translocation (75–120 mM NaCl), as observed in studies on horticultural species such as peppermint [49]. Furthermore, dill plants show strong potential for addressing Se deficiency in human diet, as demonstrated by Sheikhi et al. (2024) [14], who reported successful Se enrichment of dill microgreens in both PFAL and GH environments. Several studies have consistently shown that Se biofortification enhance the Se concentration in a wide range of fresh market products, including baby leaves, microgreens, and fruits, thereby contributing to improved dietary Se intake and consequently enhancing human health [19,20,28].

Salinity stress led to a substantial increase in the Na^+^ content of dill leaves. The excessive concentration of Na^+^ and subsequent substitution of Na^+^ for K^+^ can potentially suppress K^+^ uptake at the root level because of the antagonistic relationship between these ions at the cellular uptake sites. This is notably evident under saline conditions, where elevated Na^+^ concentrations can induce increased K^+^ leakage from cells, possibly resulting from compromised membrane integrity [61,62]. Furthermore, the relative Na^+^ and K^+^ concentrations could indicate a plant’s salinity tolerance capacity [62]. This indicator confirmed that in our study, the moderate salinity stress induced by NaCl concentration did not significantly reduce K^+^ levels in dill leaves. Supporting our findings, Ghassemi-Golezani et al. (2022) [58] demonstrated that dill plants cultivated under different salinity stress levels reported that the K^+^ concentration in leaves remained stable until the NaCl concentration reached 5 dS m^−1^ in the nutrient solution. Furthermore, the Carillo et al. (2021) [63] has reported that EC around 2 dS m^−1^ did not negatively affect the K^+^ concentration in lettuce plants cultivated in hydroponic systems with different salts (NaCl, KCl, CaCl_2_). Another significant aspect related to the K^+^/Na^+^ ratio is the role of Se biofortification in enhancing the K^+^ concentration in plants [27]. To elucidate the mechanisms by which Se mitigates the negative effects of salinity on plant growth, we investigated key physiological traits primarily affected by salinity stress.

Chl content is a key parameter for evaluating plant responses to salinity stress, and its reduction is often associated with abiotic stress conditions. A study on *Triticum durum* Desf. reported that high salinity levels negatively affect Chl synthesis and stability, mainly due to the activation of degradative enzymes such as chlorophyllase [64]. However, the ability of plants to adapt to salinity stress varies considerably among species and can be modulated by environmental and nutritional factors [4,15]. In our study, the application of moderate salinity stress did not significantly reduce Chl compared to the control conditions. In contrast, in both PFAL and GH systems, the combined treatment (Se + NaCl) resulted in an increase compared to untreated plants, suggesting that the interaction between these two factors could positively influence Chl stability [44]. This result could be attributed to the enhanced Chl biosynthesis stimulated by Se supplementation, owing to its role in transporting electrons in the respiratory chain and respiration [26]. Furthermore, Se is a key component of the antioxidant enzyme glutathione peroxidase (GSH-Px), which plays a vital role in protects cellular structures against oxidative stress by scavenging ROS [45]. This behaviour is consistent with what has been reported in the literature, where Se, under specific conditions, has been associated with maintaining photosynthetic pigment integrity under salinity stress [44]. Moreover, our results are in agreement with those obtained by Amerian et al. (2024) [44], Astaneh et al. (2019) [61], and Shekari et al. (2017) [27] found in *Cucumis sativus* L., *Allium sativum* L. and dill plants that under salinity stress conditions, Se biofortification can increase the amount of photosynthetic pigments compared to plants exposed to salinity stress alone. Moreover, the comparison between cultivation systems highlighted a generally higher chlorophyll content in plants grown in the greenhouse than in the PFAL systems, showing a consistent tendency of increased chlorophyll content under greenhouse conditions compared with indoor cultivation, as also reported by Mainos et al. (2023) [65] for rocket plants grown in different cultivation environments and seasons. Phenolics content is a useful key stress indicator in plants, in particular regarding abiotic stressors such as salinity [66]. In dill, the TPC was greater in leaves subjected to Se biofortification and moderate salinity stress than in control plants. Research indicates that Se biofortification can substantially enhance the TPC in various plant species, including brassica microgreens [67] and *Ocimum basilicum* L. plants [13], with notable increases observed in *Coriandrum sativum* L. and *Brassica rapa* subsp. *narinosa* microgreens (21% and 95%, respectively), and a confirmed boost in phenolic compounds in Brassica species. Furthermore, the TPC in dill was significantly influenced by the interplay between Se biofortification and salinity stress. In this context, the application of Se biofortification markedly increased the TPC in all dill plants across the three hydroponic systems compared to those grown under moderate salinity stress conditions alone. The potential of Se biofortification to improve TPC in crops facing salinity stress has been previously recognised in important horticultural plants such as brassicas [67] and *Allium sativum* L. [61]. Dill plants grown in the three hydroponic systems exhibited higher phenolic concentrations under GH conditions than under PFAL systems. This result is consistent with previous studies on red *L. sativa* L. [68] and *Brassica oleracea* var. italica microgreens [69], which reported higher phenol concentration in GH crops than in indoor crops. This increase can be attributed to full sunlight exposure, which includes the UV component and temperature variations, both of which can stimulate phenolic synthesis [70,71]. In indoor farms, the absence of UV light and constant temperature probably reduced this adaptive response, confirming the influence of environmental conditions on the nutritional quality of plants.

Salinity is a significant abiotic stress that limits plant growth and productivity worldwide, primarily by inducing oxidative stress through the overproduction of ROS, which damage membranes, proteins, lipids, and nucleic acids [72,73]. In response, plants activate both non-enzymatic (e.g., phenolic compounds) and enzymatic antioxidant defences, such as APX, CAT, and GPX, to regulate ROS and limit oxidative damage. Recent studies have highlighted Se as a promising agent for enhancing these antioxidant systems under salinity. Se supplementation has been shown to boost APX, CAT, and GPX activities, contributing to improved ROS detoxification and cellular protection in various species [48,74,75]. Various studies have also revealed that Se can modulate the expression of genes related to antioxidant defence pathways and act as a signalling agent in stress responses, leading to increased synthesis of secondary metabolites, such as flavonoids and TPC [76]. This study indicated that the antioxidant effect of Se was associated with the accumulation of antioxidant compounds required to preserve cell membrane integrity under salinity stress. This relationship reflects the specific role of Se in enhancing the antioxidant system’s capacity to scavenge reactive oxygen species (ROS), thereby reducing oxidative damage. Se contributes to maintaining redox homeostasis by stimulating the activity of key antioxidant enzymes, such as superoxide dismutase (SOD), CAT, and GPX, which collectively limit the accumulation of ROS like H_2_O_2_ within chloroplasts and other cellular compartments. In our experiments, the activity of these oxidative enzymes was higher in dill plants cultivated with Se biofortification under moderate salinity stress than in plants subjected to control conditions or Se biofortification alone, suggesting a synergistic effect between Se and salinity in enhancing the plant’s antioxidant defence. This finding is consistent with previous observations that Se application minimizes H_2_O_2_ levels in chloroplasts [76]. Several research works have documented this phenomenon, demonstrating that plants such as *Setaria italica* L. [77] and *Mentha suaveolens* Ehrh. [75] exposed to both Se and salinity stress showed a synergistic effect, with elevated enzymatic responses compared to individual treatments. Various horticultural species, including. *Ocimum basilicum* L., have exhibited marked improvements in their antioxidant capabilities in response to Se biofortification, highlighting its role as a potent stimulator of plant defense mechanisms [78]. These improvements manifest as enhanced enzymatic activities and increased synthesis of non-enzymatic antioxidants, strengthening the plant’s resistance to oxidative damage under saline conditions. The protective effects of Se are known to be concentration-dependent, with optimal doses varying among plant species and cultivars; in this study, the use of 5 μM Se likely provided a level sufficient to trigger beneficial responses without causing toxicity. The observed variations in enzymatic activities between different growing conditions, such as PFAL versus GH, underscore the potential influence of environmental factors on plant responses to abiotic stress, particular the quality and intensity of light and temperature [79,80]. These findings suggest that integrating Se biofortification with optimized environmental conditions could enhance the physiological mechanisms underlying salinity tolerance, such as improved antioxidant defense, osmotic adjustment, and membrane stability, thus representing a promising strategy to sustain crop performance and quality under-round cultivation in CEA systems.

## 4. Materials and Methods

### 4.1. Experiment 1: PFAL Cultivation

*Experimental site and design.* The first experiment (Exp1) was conducted at the Department of Agricultural, Forest and Food Sciences (DISAFA), University of Turin, Grugliasco, Italy (45°03′58″ N; 7°35′20″ E; 294 m a.s.l.) growing dill (*A. graveolens* L.) with two hydroponic systems. Two independent replications were conducted in 2023, each for 23 days. A randomised complete block design (RCBD) was adopted, with two runs considered as replications (blocks). Each replication included all four treatments: without Se biofortification and moderate salinity stress (Control), foliar Se biofortification (Se), moderate salinity stress (NaCl), and combined Se biofortification and moderate salinity (Se + NaCl). Each treatment consisted of 480 plants (experimental unit).

**Figure 3:**
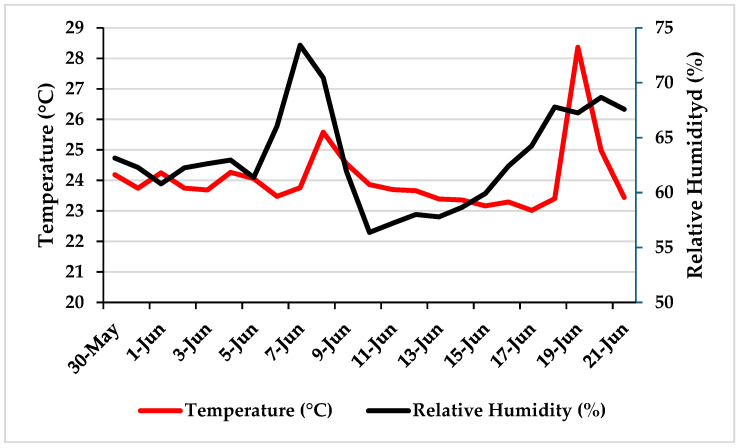
Temperature and relative humidity recorded in the experimental PFAL during the trial.

*Cultivation systems and PFAL setup*. Dill plants were grown in a multilayer vertical hydroponic system “Radix”(Fujian Sananbio Technology Co., Ltd., Xiamen, China) equipped with both a floating system (FS; 30 L capacity) and a nutrient film technique (NFT; 15 L capacity), with gravity-driven water circulation layers into the growing beds (total cultivable area = 4.2 m^2^; internal volume = 2.62 m^3^). The module was fully automated using a NIDO ONE V1 hydroponic controller (Nido S.r.l., Carpineti, RE, Italy) that every 15 min monitored and managed air temperature, relative humidity (RH) (Figure 3), pH, electrical conductivity (EC), and nutrient solution volume.

*Environmental conditions and lighting.* The average environmental conditions during cultivation were 24.3 °C and 65% RH (Figure 3). Lighting was provided by Sananbio “Vegmax” LEDs (61% red, 13% blue, 15% green, 11% far-red; red:blue ratio = 5.08), with a PPFD of 255 µmol m^−2^ s^−1^ at the canopy level, a photoperiod of 14 h (06:00–20:00), and a lamp-to-canopy distance of 30 cm.

*Nutrient solution management and monitoring.* The nutrient solution was prepared with 12 mM N (NO_3_^−^:NH_4_^+^ = 60:40), 2 mM P, 6 mM K^+^, 2 mM Mg^2+^, and 2.5 mM Ca^+^. Micronutrients were supplied as follows (in mM): 0.14 B, 0.05 Cu, 0.11 Fe, 0.22 Mn, 0.002 Mo, and 0.14 Zn. Lysodin^®^ Multimix (Intrachem Production S.r.l., Grassobbio, BG, Italy) was added at 0.30 g L^−^ [81]. For salinity stress treatments, the addition of NaCl resulted in a concentration of 10 mM of both Na^+^ and Cl^−^ ions (Na^+^:K^+^ ratio = 1.67:1; EC ~2.8–3.0 dS m^−1^), compared to control (Na^+^:K^+^ ratio <0.08:1; EC ~1.6–2.0 dS m^−1^). The pH of the solution was maintained between 5.5 and 6.0, and EC was 1.6 dS m^−1^ in the control and Se treatments and 2.0 dS m^−1^ in the NaCl and Se + NaCl treatments. Salinity stress was imposed by supplementing the nutrient solution with NaCl to reach 10 mM NaCl final concentration. The system allowed real-time monitoring and immediate correction of deviations from the predefined target ranges (pH 5.5–6.0; for control EC ~1.6–2.0 dS m^−1^ for salinity stress treatments EC ~2.8–3.0 dS m^−1^, depending on treatment). When values deviated from the target ranges, pH was adjusted automatically by the NIDO controller using 20% (*v*/*v*) phosphoric acid, and EC was restored by adding fresh nutrient solution to the original level.

*Seedling production, uniformity criteria, and transplanting.* Seeds were supplied by “Azienda Agricola di Ricca Sebastiano” (Carignano, TO, Italy) and sown in rockwool plugs (25 × 25 × 40 mm; Grodan^®^ Delta, Grodan BV, Roermond, The Netherlands), four seeds per plug, and incubated in darkness for three days at 23 °C. The seedlings were then transferred to the light regime described above and irrigated twice daily for 1 min until transplantation. Eight days after sowing, uniform seedlings with four true leaves in rockwool plugs were transplanted into Radix FS trays measuring with a plant density of 1600 plants m^−2^. Six days after transplanting, NaCl treatments were added to NS for NaCl and Se + NaCl treatments.

*Selenium biofortification treatment and harvest.* Foliar Se biofortification was carried out once, seven days after transplanting, by spraying 5 μM sodium selenate (Na_2_SeO_4_, purity ≥ 99.8%; Thermo Scientific GmbH, Darmstadt, Germany) uniformly across all leaves at 09:00 h. Each dill tray received 37 mL of Se solution, distributed evenly, resulting in approximately 0.077 mL of sodium selenate solution per plant, which contained 0.0728 μg of sodium selenate [81]. Control and NaCl treatments were administered distilled water. Harvesting was conducted 23 days after sowing, corresponding to 15 days after transplanting and one week after foliar Se biofortification. All 480 plants in each bed were harvested together for biomass determination. To minimize potential edge effects, plants from the central portion of each bed were randomly selected for the analytical determinations.

### 4.2. Experiment 2: Greenhouse Cultivation

*Experimental site and design.* The second experiment (Exp2) was conducted at the DISAFA Agricultural and Livestock Experiment Station “Tetto Frati” (44°53′11.67″ N; 7°41′7.00″ E; 231 m a.s.l., Carmagnola, TO, Italy) from 9 June to 7 July in a glasshouse. An RCBD was adopted with three benches used as replications (blocks), each divided into four flotation beds (2.50 × 1.40 × 0.15 m; 200 L per bed), corresponding to the four treatments of the floating system (FS) adopted: Control, Se, NaCl, and Se + NaCl [82]. Each treatment consisted of 480 plants (experimental unit).

**Figure 4:**
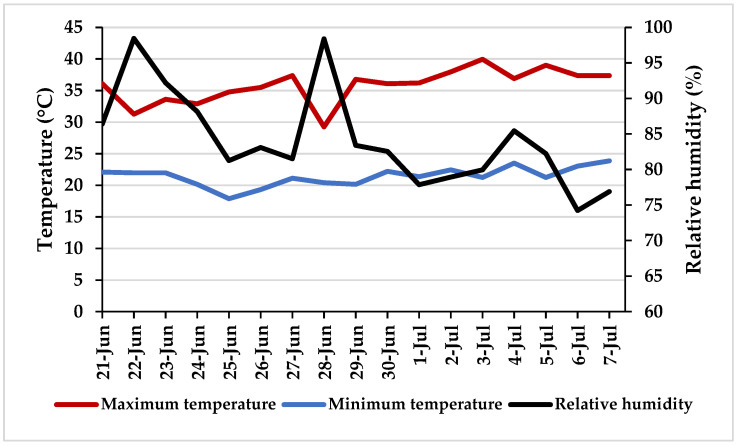
Temperatures and relative humidity recorded in the experimental greenhouse during the trial.

*Greenhouse Environmental Conditions*. The greenhouse was equipped with an automatically controlled ventilation and temperature system. During the cultivation period, the maximum, minimum, and mean air temperatures were 36.1 °C, 21.7 °C, and 28.0 °C, respectively, with an average relative humidity (RH) of 82% (Figure 4).

*Nutrient solution management and monitoring.* The FS nutrient solution was continuously aerated, and pH (5.5–6.0) and EC (1.6 dS m^−1^ in Control and Se treatments and 2.8–3.0 dS m^−1^ in treatments NaCl and NaCl + Se, respectively) were monitored manually every two days using a portable conductivity and pH meter (CyberScan PC 650, Eutech Instruments, Singapore; www.eutechinst.com) with immediate correction of deviations from the predefined target ranges (pH 5.5–6.0; for control EC ~1.6–2.0 dS m^−1^ for salinity stress treatments EC ~2.8–3.0 dS m^−1^, depending on treatment). When values deviated from the target ranges, pH was adjusted manually using 20% (*v*/*v*) phosphoric acid, and EC was restored by adding fresh nutrient solution to the original level. The same nutrient solution described in Experiment 1 was used to fill the FS beds in this experiment.

*Seedling production, uniformity criteria, and transplanting.* Dill seeds, supplied by the same company as in Experiment 1, were sown in 60 cell polystyrene trays (four seeds per cell; ca. 240 plants tray^−1^) filled with a peat-based horticultural medium (Neuhaus Huminsubstrat N17; Klasmann Deilmann^®^ GmbH, Geeste, Germany). Trays were incubated in a germination chamber (23 °C; 95% RH; dark) for three days and then transferred to a plastic greenhouse for nine days, irrigated twice daily for 1 min per event. Twelve days after sowing, trays with seedlings at the four true leaf stage were transplanted into the FS beds at a density of approximately 1562 plants m^−2^.

*Selenium biofortification treatment and harvest.* Twelve days after transplanting, salinity treatments were imposed by adding 10 mM NaCl to the nutrient solution of the respective beds. One day later (13 d after transplanting), foliar Se biofortification was carried out with 5 μM Na_2_SeO_4_, as described for Experiment 1. Harvesting was performed 32 days after sowing (20 days after transplanting and 7 days after Se biofortification), when plants reached a canopy height of approximately 25 cm. All 480 plants in each bed were harvested together for biomass determination. To minimize potential edge effects, plants from the central portion of each bed were randomly selected for the analytical determinations.

### 4.3. Plant Growth Measurements

At harvest, the canopy from each replicate was washed and dried with blotting paper, and the yield was recorded in g m^−2^. A subsample of leaves (10 g) was immediately frozen in liquid nitrogen and stored at –80 °C for further physiological and biochemical analyses. To determine the DW, fresh samples were oven-dried at 60 °C (Binder ED56, Binder GmbH, Tuttlingen, Germany) until a constant weight was reached.

### 4.4. Total Chlorophyll Content

Total Chl was analyzed by grinding fresh leaves (300 mg per replicate) in liquid nitrogen to ensure complete cellular disruption. The ground tissue was mixed with 15 mL of high-purity ethanol (96% *v*/*v*) and stored at 4 °C in the dark for 24 h. The extracts were filtered and analyzed spectrophotometrically using a Cary spectrophotometre (Agilent Technologies, Santa Clara, CA, USA) at 665 nm for chlorophyll a (Chl a) and 649 nm for chlorophyll b (Chl b). Chl a and Chl b concentrations were calculated using the formulas from Lichtenthaler & Wellburn (1983) [83] and expressed as mg g^−1^ fresh weight (FW).

### 4.5. Extraction and Measurement of Total Phenolics Content

Fresh leaves (1 g per replicate) were crushed in a mortar and extracted with methanol (1:3, *w*/*v*) and liquid nitrogen. The extracts were kept in an ice bath for 30 min and centrifuged at 5000× *g* for 30 min at 4 °C. The supernatant was stored at −20 °C until analysis. The TPC was measured using a modified version of the method described by Arnaldos et al. (2002) [84]. The phenolic extract (100 µL), 2% sodium carbonate (Na_2_CO_3_; 1 mL), and Folin-Ciocalteau reagent (75 µL; Sigma-Aldrich; Saint Louise, MO, USA) were added to the phenolic extract. After incubation for 15 min at 25 °C in the dark, the absorbance was measured spectrophotometrically at 725 nm. Gallic acid was used as a standard [85].

### 4.6. Enzyme Extraction and Assay Conditions

Fresh green leaves (0.1 g per replicate) were extracted with a solution containing 62.5 mM potassium phosphate (KH_2_PO_4_), as reported by Ertani et al. (2013) [86]. The homogenates were centrifuged, and the supernatants were used immediately for enzymatic activity determination.

APX (EC 1.11.1.11) activity was evaluated using an extraction mixture that consisted of 50 mM potassium phosphate buffer (pH 7.0), 1 mM EDTA Na_2_, 0.5 mM ascorbic acid, 0.1 mM hydrogen peroxide (H_2_O_2_), and 50 µL of crude enzymatic extract. APX enzyme activity was evaluated by monitoring the decrease in ascorbate (extinction coefficient of 2.8 mM^−1^ cm^−1^) and measuring the absorbance spectrophotometrically at 290 nm over a 2 min interval [86].

CAT (EC 1.11.1.6) activity was evaluated using a reaction mixture containing 50 mM phosphate buffer (pH 7.0), 20 mM polyvinylpyrrolidone (PVP), and 250 µL Triton X-100. The reaction mixture contained 64 mM KH_2_PO_4_, 10 mM H_2_O_2_, and 50 µL of enzyme extract. CAT activity was determined by examining the consumption of H_2_O_2_ (extinction coefficient of 39.4 mM^−1^ cm^−1^) spectrophotometrically (Cary spectrophotometer, Agilent Technologies, Santa Clara, CA, USA) at 240 nm over a 2 min interval [86].

GPX (EC 1.11.1.7) activity was evaluated with a mixture of 0.05 mL guaiacol (20 mM), 2.9 mL potassium phosphate buffer (10 mM, pH 7.0), and 50 µL of the enzyme. The reaction was initiated by adding 2 mL of 40 mM H_2_O_2_ GPX enzyme activity was evaluated by measuring the oxidation of guaiacol in the presence of H_2_O_2_ (extinction coefficient 26.6 mM^−1^ cm^−1^) spectrophotometrically at 470 nm over a 2 min interval [86].

### 4.7. Selenium, Potassium, Sodium, and Magnesium Content

Dried leaves (1.5 g per replicate) were prepared by grinding and drying, followed by the mineralisation procedure. The digestion process included the addition of 7 mL of concentrated nitric acid (70% *v*/*v* HNO_3_) and 1.5 mL of hydrogen peroxide (30% *v*/*v* H_2_O_2_) to facilitate complete sample digestion. Mineralisation was carried out using an Ethos 1 microwave digestion system (Milestone Inc., Shelton, CT, USA), which ensured thorough and consistent sample preparation.

Elemental analyses of Se, K^+^, Na^+^, and Mg^2+^ were performed using an inductively coupled plasma mass spectrometer (ICP-MS) Xseries II (Thermo Scientific Inc., Bremen, Germany), following the methodological approach described by Squadrone et al. (2016, 2017) [87,88]. To ensure analytical reliability, the method’s recovery was verified using a standard reference material (SRM 1573a, Tomato Leaves, National Institute of Standards and Technology) and a certified reference material (BCR ^®^-668, Joint Research Centre). The limit of quantification (LOQ) was set at 0.010 mg kg^−1^ for trace elements and 0.001 mg kg^−1^ for rare earth elements (REEs) [88].

### 4.8. Nitrogen, Carbon and Sulphur Content

Using an UNICUBE elemental analyser (Elementar Analys system GmbH, Langenselbold, Germany), 60 mg of dried leaves per replicate were weighed and placed in 90 mg tungsten capsules. The capsules were sealed and inserted into the instrument after the air was removed. The analysis involved several steps, as follows. First, the sample was burned at 1150 °C. Oxygen was added in two steps: 30 mL L^−1^ for 30 s and then 100 mL L^−1^ for 120 s. The sample passed through a tube with CuO and Pt catalysts, converting all parts into gases such as nitrogen oxides (NOx), carbon dioxide (CO_2_), and sulphur dioxide (SO_2_). Helium gas was used to transport the gases to the detector. The detector uses a method to separate and identify gases. NOx was detected first, whereas CO_2_ and SO_2_ were retained on dedicated heated columns. The CO_2_ column was heated to 230 °C to release and detect CO_2_. The SO_2_ column was heated to 210 °C to detect SO_2_. The amounts were measured using a calibration curve with sulphanilamide (N = 16.23%, S = 18.61%, and C = 41.62%).

### 4.9. Statistical Analysis

Data from each cultivation system were submitted to analysis of variance (ANOVA) for the treatments. Data from all cultivation systems, using mean values averaged across treatments, were submitted to ANOVA for the systems. In Exp1 and Exp2, RCBD was used with two and three replications, respectively. Comparisons of means were made using the Bonferroni post hoc test (*p* ≤ 0.05), and the results are presented as mean ± standard error (SE). Statistical analyses were performed in R using the RStudio integrated development environment (version 2023.12.1). Linear models and one-way of ANOVA were conducted using the base *stats* package, while post hoc comparisons were performed using the *emmeans* and *multcomp* packages with Bonferroni adjustment. Pearson’s two-tailed correlation test was employed to examine the relationships between the variables. Pearson’s two-tailed correlation test and principal component analysis (PCA) were performed using the XLSTAT software (version 2018.1, Addi soft, Paris, France).

## 5. Conclusions

This study evaluated the effects of Se biofortification and moderate salinity stress on dill grown in PFAL and GH systems. The findings demonstrated that the combined application of Se (5 μM) and moderate salinity stress (10 mM NaCl) did not negatively affect yield or DW, confirming that these conditions are non-toxic and do not compromise productivity. Moreover, Se biofortification combined with moderate salinity stress consistently resulted in higher Se concentration in dill plants than Se biofortification alone. This effect was observed across all tested hydroponic systems (NFT-PFAL, FS-PFAL, and FS-GH), indicating that the presence of NaCl help maintain Se uptake regardless of the cultivation environment. A significant increase in Se concentration was detected in plants grown under PFAL conditions, suggesting that the strict environmental control typical of indoor promotes greater micronutrient accumulation. Concurrently, the combined treatment maintained, or in some cases enhanced, Chl content and stimulated the synthesis of TPC. These physiological responses were accompanied by the activation of antioxidant enzymes, including APX, CAT, and GPX, which strengthened the plants’ defence mechanisms against oxidative stress. Overall, the combined application of Se biofortification and moderate salinity stress represents a promising strategy to enhance the nutritional quality of dill by increasing Se concentration. While the PFAL system allows for precise environmental control, leading to faster growth and higher Se accumulation, the GH system benefits from natural light, which favours higher synthesis of TPC and Chl. Together, these findings highlight the potential of integrating Se biofortification into CEA systems to improve dietary Se intake and contribute to human health. Future studies should explore a broader range of Se concentrations and salinity levels, as well as extend this approach to other aromatic and medicinal species, while also addressing the role of planting density, cultivation duration, and system-specific environmental drivers (e.g., light quality and atmospheric demand) to further refine the interpretation of yield stability and dry matter accumulation in sustainable food production.

## Figures and Tables

**Table 1:** Effects of foliar-applied selenium (Se) under salinity stress on yield, dry weight, total chlorophyll content, total phenol content, guaiacol peroxidase, catalase, and ascorbate peroxidase of dill cultivated in three hydroponic systems.

Treatments	Yield	Dry Weight	Total Chlorophyll Content	Total Phenol Content	Guaiacol Peroxidase	Catalase	Ascorbate Peroxidase
	g m^−2^		mg g^−1^ (FW)	nmol mg^−1^ (FW)	μmol min^−1^ mg^−1^ protein		
Dill NFT-PFAL							
Control	1701.55 ± 75.5	160.32 ± 4.96	4.24 ± 0.02 a	1.85 ± 0.01 d	95.65 ± 0.49 c	28.62 ± 0.12 b	34.21 ± 0.10 d
Se	1899.13 ± 42.26	169.94 ± 6.82	3.08 ± 0.01 c	3.28 ± 0.01 c	111.56 ± 0.56 b	28.32 ± 0.30 b	38.76 ± 0.01 c
NaCl	1827.00 ± 48.33	170.9 ± 9.41	2.47 ± 0.01 d	4.11 ± 0.02 b	113.2 ± 1.64 b	37.59 ± 1.01 a	39.47 ± 0.11 b
Se + NaCl	1813.01 ± 23.00	178.71 ± 5.88	3.36 ± 0.03 b	4.88 ± 0.01 a	131.25 ± 2.18 a	41.77 ± 1.42 a	46.33 ± 0.08 a
Significance	ns	ns	***	***	***	***	***
Dill FS-PFAL							
Control	1819.91 ± 42.83	168.26 ± 5.81	5.47 ± 0.01 a	1.47 ± 0.01 c	131.29 ± 1.64 b	28.59 ± 0.01 b	34.1 ± 0.37 c
Se	1810.16 ± 35.30	159.31 ± 3.17	3.08 ± 0.01 c	2.79 ± 0.02 b	135.62 ± 2.18 b	28.83 ± 0.08 b	40.42 ± 0.03 b
NaCl	1881.33 ± 24.00	160.81 ± 1.75	2.48 ± 0.02 d	4.66 ± 0.05 a	141.09 ± 3.28 a	35.37 ± 0.62 a	43.61 ± 0.43 a
Se + NaCl	1854.20 ± 38.86	179.84 ± 2.02	3.94 ± 0.01 b	4.75 ± 0.01 a	147.65 ± 3.18 a	36.46 ± 0.88 a	43.47 ± 0.03 a
Significance	ns	ns	***	***	***	***	***
Dill FS-GH							
Control	1872.54 ± 155.95	202.83 ± 5.46	4.70 ± 0.91	3.62 ± 0.05 c	156.77 ± 9.57 c	29.12 ± 0.19 b	43.16 ± 0.93 b
Se	1980.39 ± 157.41	176.25 ± 12.01	4.16 ± 0.36	3.66 ± 0.33 c	182.29 ± 3.64 b	29.25 ± 0.04 b	45.02 ± 0.13 b
NaCl	1821.35 ± 54.75	185.40 ± 5.59	3.88 ± 0.23	5.14 ± 0.40 b	266.87 ± 1.89 a	42.94 ± 1.78 a	48.51 ± 0.87 a
Se + NaCl	1859.08 ± 129.34	190.41 ± 14.67	4.89 ± 0.38	6.63 ± 0.18 a	246.79 ± 6.05 a	43.04 ± 1.77 a	49.27 ± 0.54 a
Significance	ns	ns	ns	***	***	***	***

Different letters within columns indicate significant differences (*p* ≤ 0.05) among the treatments based on the Bonferroni test. Control; NaCl (sodium chloride at 10 mM); Se (5 μM); Se + NaCl. Mean ± standard error. *** = *p* ≤ 0.001, ns = non-significant.

**Table 2:** Effects of three hydroponics systems on yield, dry weight, total chlorophyll content, total phenol content, guaiacol peroxidase, catalase, and ascorbate peroxidase during dill cultivation.

Dill_Soilless Cultivation	Yield	Dry Weight	Total Chlorophyll Content	Total Phenol Content	Guaiacol Peroxidase	Catalase	Ascorbate Peroxidase
	g m^−2^		mg g^−1^ (FW)	nmol mg^−1^ (FW)	μmol min^−1^ mg^−1^ protein		
NFT-PFAL	1808.65 ± 33.21	169.97 ± 3.60 b	3.29 ± 0.24 b	3.53 ± 0.42	112.77 ± 4.80 b	34.08 ± 2.21	39.69 ± 1.63 b
FS-PFAL	1841.40 ± 17.28	166.55 ± 3.08 b	3.74 ± 0.42 ab	3.42 ± 0.51	121.54 ± 13.17 b	32.31 ± 1.38	40.40 ± 1.46 b
FS-GH	1883.52 ± 58.69	188.72 ± 5.24 a	4.41 ± 0.26 a	4.76 ± 0.39	211.43 ± 14.97 a	36.08 ± 2.14	46.49 ± 0.81 a
Significance	ns	**	*	ns	***	ns	***

Different letters within columns indicate significant differences (*p* ≤ 0.05) among the treatments based on the Bonferroni test. NFT-PFAL (Nutrient Film Technique-Plant Factory with Artificial Lighting); FS-PFAL (Floating Systems-Plant Factory with Artificial Lighting); FS-GH (Floating System-Greenhouse). Mean ± standard error. *** = *p* ≤ 0.001, ** = *p* ≤ 0.01, * = *p* ≤ 0.05, ns = non-significant.

**Table 3:** Effects of Se and salt stress treatments on the Se, Na^+^, K^+^, Mg^2+^, N, C, and S concentrations of dill cultivated in three hydroponics systems.

Treatment	Se	Na^+^	K^+^	Mg^2+^	N	C	S
	mg kg^−1^ (DW)	mg kg^−1^ (DW)	g kg^−1^ (DW)	g kg^−1^ (DW)	(%)	(%)	(%)
Dill NFT-PFAL							
Control	0.58 ± 0.09 b	437.20 ± 31.69 b	66.71 ± 1.17 a	5.73 ± 0.17	6.16 ± 0.17	40.54 ± 0.04	0.63 ± 0.05
Se	25.55 ± 4.14 b	388.75 ± 18.98 b	68.56 ± 0.20 a	5.49 ± 0.30	6.18 ± 0.02	40.66 ± 0.55	0.70 ± 0.02
NaCl	0.58 ± 0.01 b	680.75 ± 55.75 a	54.66 ± 0.99 b	5.72 ± 0.39	5.97 ± 0.38	39.51 ± 1.07	0.66 ± 0.03
Se + NaCl	31.78 ± 1.02 a	616.15 ± 12.92 ab	57.10 ± 1.57 b	6.04 ± 0.21	6.10 ± 0.02	40.83 ± 0.35	0.62 ± 0.03
Significance	***	**	**	ns	ns	ns	ns
Dill FS-PFAL							
Control	0.86 ± 0.03 b	419.74 ± 30.4 b	64.78 ± 1.28	5.47 ± 0.15 b	5.83 ± 0.07	40.42 ± 0.87	0.60 ± 0.00
Se	23.67 ± 1.30 a	337.03 ± 1.64 b	63.38 ± 0.60	5.22 ± 0.14 b	5.85 ± 0.05	40.54 ± 0.41	0.68 ± 0.02
NaCl	0.66 ± 0.11 b	595.88 ± 0.96 a	59.51 ± 1.63	6.30 ± 0.02 a	5.92 ± 0.11	40.46 ± 0.09	0.65 ± 0.03
Se + NaCl	33.12 ± 4.57 a	605.30 ± 6.77 a	62.04 ± 2.15	5.88 ± 0.01 ab	5.89 ± 0.01	39.19 ± 0.03	0.57 ± 0.01
Significance	**	***	ns	*	ns	ns	ns
Dill FS-GH							
Control	0.70 ± 0.13 c	1260.39 ± 150.25 b	57.97 ± 4.07	4.36 ± 0.46	5.56 ± 0.03	38.56 ± 1.41	0.87 ± 0.09
Se	17.83 ± 1.02 b	1060.52 ± 28.33 b	53.38 ± 3.62	4.50 ± 0.11	5.55 ± 0.01	39.22 ± 1.95	0.97 ± 0.15
NaCl	0.60 ± 0.13 c	2427.12 ± 85.69 a	51.34 ± 3.27	4.83 ± 0.65	5.53 ± 0.04	40.64 ± 0.70	0.84 ± 0.07
Se + NaCl	23.32 ± 0.59 a	2367.34 ± 85.30 a	52.42 ± 3.71	4.89 ± 0.20	5.55 ± 0.05	38.48 ± 1.79	1.11 ± 0.09
Significance	***	***	ns	ns	ns	ns	ns

Different letters within columns indicate significant differences (*p* ≤ 0.05) among the treatments based on the Bonferroni test. Control; NaCl (sodium chloride at 10 mM); Se (selenium at 5 μM); Se + NaCl. Mean ± standard error. *** = *p* ≤ 0.001, ** = *p* ≤ 0.01, * = *p* ≤ 0.05, ns = non-significant.

**Table 4:** Effects of three hydroponic systems on the content of Se, K, Na^+^, K^+^, Mg^2+^, C, and S concentrations of dill.

Dill Soilless Cultivation	Se	Na^+^	K^+^	Mg^2+^	N	C	S
	mg kg^−1^ (DW)	mg kg^−1^ (DW)	g kg^−1^ (DW)	g kg^−1^ (DW)	(%)	(%)	(%)
NFT-PFAL	14.62 ± 5.43 a	530.71 ± 47.56 b	61.76 ± 2.29 a	5.74 ± 0.13 a	6.10 ± 0.08 a	40.39 ± 0.30	0.66 ± 0.01 b
FS-PFAL	14.58 ± 5.44 a	489.49 ± 43.84 b	62.43 ± 0.93 a	5.72 ± 0.16 a	5.87 ± 0.03 b	40.15 ± 0.27	0.63 ± 0.01 b
FS-GH	10.61 ± 3.07 b	1778.84 ± 192.31 a	53.78 ± 1.74 b	4.65 ± 0.19 b	5.55 ± 0.01 c	39.22 ± 0.70	0.95 ± 0.05 a
Significance	**	***	**	***	***	ns	***

Different letters within columns indicate significant differences (*p* ≤ 0.05) among the treatments based on the Bonferroni test. NFT-PFAL (Nutrient Film Technique-Plant Factory with Artificial Lighting); FS-PFAL (Floating Systems-Plant Factory with Artificial Lighting); FS-GH (Floating System-Greenhouse). Mean ± standard error. *** = *p* ≤ 0.001, ** = *p* ≤ 0.01, ns = non-significant.

## Data Availability

The raw data supporting the conclusions of this article will be made available by the authors on request.
